# Effects of public–private partnership on diet-related obesity risk factors among school-aged children: A systematic literature review

**DOI:** 10.1177/02601060221136184

**Published:** 2022-11-04

**Authors:** Megan R Harrison

**Affiliations:** 4906 London School of Hygiene & Tropical Medicine, London, UK

**Keywords:** public–private partnership, PPP, childhood obesity, overweight, school-aged children

## Abstract

**Background:** Childhood obesity is a major public health challenge. Public–private partnerships (PPPs) have been proposed as a solution; however, valid concerns exist as to whether commercial interest can be balanced with public health goals. **Aims:** This study describes the effects of interventions carried out through PPPs on diet-related obesity risk factors, namely fruit and vegetable (F&V), sugar-sweetened beverage (SSB), and energy-dense food consumption, among school-aged children. **Methods:** A systematic literature review was conducted from January 1990 to December 2021 across three databases. Out of the 276 articles initially identified, 8 were included. Data were extracted from each article on study characteristics, partners involved, partnership descriptions, and partnership outcomes. A descriptive analysis included frequency counts for specific study attributes. **Results:** All studies took place in the United States and were published between 2010 and 2017. Most were cohort studies (75%) and involved structured, healthy lifestyle interventions (75%). Nearly all interventions included components targeting F&V consumption (88%), followed by energy-dense food consumption (50%), and SSB consumption (38%). Business sector partners were largely food producers, food retailers, and private healthcare providers; however, few studies provided details on their partnering arrangements. No studies reported harmful changes in diet-related obesity risk factors. **Conclusion:** Collaboration across sectors is needed to address drivers of obesity where children live, learn, and play. The small sample size and heterogeneity in this review prohibits definitive conclusions pertaining to the effect of PPPs on childhood obesity. Future research efforts are needed to develop a taxonomy for better classifying and examining PPPs.

## Introduction

Childhood obesity is one of the major global public health challenges of the 21st century. In 2016, approximately one in five children aged 5–19 years were overweight and 6.8% were living with obesity (World Health Organization; World Health Organization, 2019). Levels are continuing to rise around the world as demonstrated by a recent global analysis showing the prevalence of obesity rose eightfold among girls and nearly ninefold among boys aged 5–19 years from 1975 to 2016 (Abarca-Gómez et al., 2017). Obesity during childhood poses immediate health risks (Cote et al., 2013; Lloyd et al., 2012; Bacha and Gidding, 2016; Di Bonito et al., 2018), and often persists into adulthood (Gordon-Larsen et al., 2010) where it is associated with increased risk of major non-communicable diseases (NCDs) including coronary heart disease, stroke, type 2 diabetes, and several types of cancer (Guh et al., 2009; Lauby-Secretan et al., 2016). While the drivers of obesity are complex, poor diet is a leading risk factor. In 2017, unhealthy diets were found to be responsible for more deaths globally than any other risk factor (Afshin et al., 2019). Unhealthy diets are generally those low in fibers, fruits, vegetables, legumes, whole grains, nuts and seeds, milk, seafood, calcium, and healthy fats (omega 3 fatty acids and polyunsaturated fatty acids) and high in *trans*-fatty acids, sodium, red or processed meat, and sugar-sweetened beverages (SSBs) (Afshin et al., 2019).

Spurred by the complex nature of obesity, intersectoral partnerships have been promoted as an important tool for responding to the childhood obesity epidemic (Hendriks et al., 2013; Institute of Medicine, 2015). As the understanding of drivers of childhood obesity has expanded, so too has the need for a diverse range of expertise and skill sets to address the public health problem. Intersectoral partnerships bring together different sectors of society (e.g. healthcare, education, housing, law enforcement, agriculture, transportation etc.) to collaborate on a shared issue. *Public–private partnership* (PPP) is an umbrella term used to describe the continuum of intersectoral partnering arrangements between governmental and private sector entities for the purpose of supporting governments’ broader service responsibilities (World Bank, 2017; Roehrich et al., 2014). The term rarely appeared in the literature prior to the 1990s, but has grown exponentially due to (i) skepticism of private sector-led solutions, (ii) a growing pattern of collaboration with national governments, and (iii) investments by high-net-worth individuals (Roehrich et al., 2014; Barr, 2007). Today, PPPs are promoted as a critical component of the Sustainable Development Agenda 2030 through Goal 17 (United Nations Sustainable Development Goals, 2015), which aims to “Encourage and promote effective public, public-private and civil society partnerships, building on the experience and resourcing strategies of partnerships” (p. 1).

Proponents of addressing childhood obesity through PPPs argue the issue is too complex to be addressed by governments alone (Institute of Medicine, 2012), that PPPs play a vital role in modifying the food supply (Institute of Medicine, 2012; Fanzo et al., 2021), are important for promoting health in all policies (Hendriks et al., 2013), contribute important shared resources and expertise (Institute of Medicine, 2012), and can be instrumental for driving research agendas (Perry et al., 2015). Critics of PPPs have focused largely on the conflicts of interest introduced when public organizations partner with businesses motivated by commercial interests (Institute of Medicine, 2012; Fanzo et al., 2021; Ralston et al., 2020). Conflicts of interest may be irreconcilable depending on the initiative and partners involved. For example, in addressing childhood obesity, it would be unlikely for a health promotion organization to maintain credibility while also partnering with an organization known to use aggressive and predatory marketing tactics toward young children (Institute of Medicine, 2012). However, other risks entailed with PPPs includ information sharing outside the partnership, poor participation in the partnership, concerns pertaining to endorsement perceptions, and undue influence over the scientific process (Institute of Medicine, 2012).

To the author's knowledge, no previous reviews of the PPP literature pertaining to childhood obesity have been undertaken. The purpose of this review was to describe the effects of interventions carried out through PPPs on diet-related obesity risk factors among school-aged children.

## Theoretical background

### Defining PPPs

**Figure 1.**
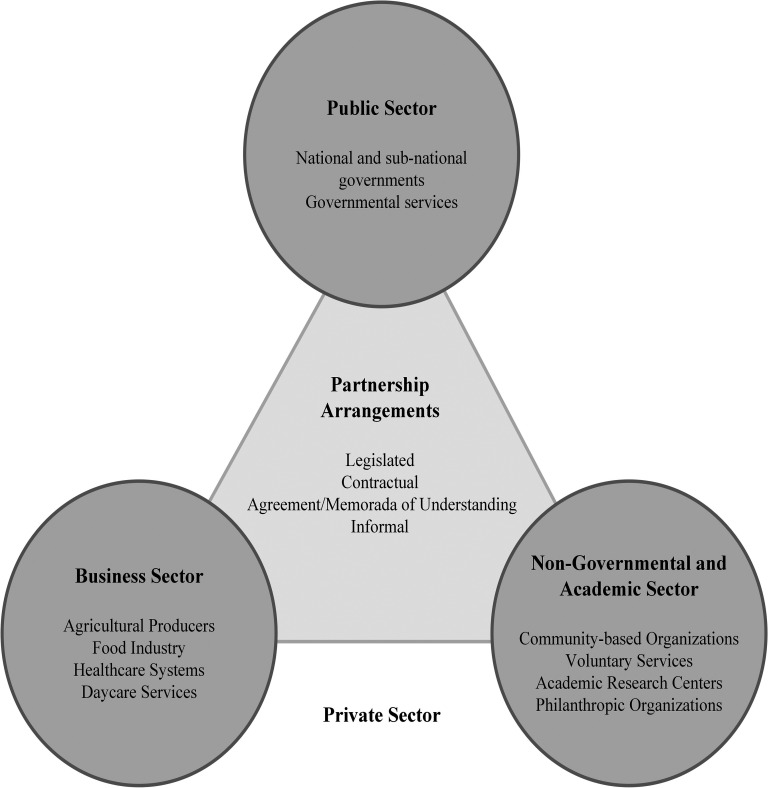
Conceptual model of public–private partnerships (PPP) for childhood obesity prevention and management.

Adapted from Bajracharya B and Hastings P (2015). Public-private partnerships in emergency and disaster management: Examples from the Queensland floods 2010–2011. The Australian Journal of Emergency Management ;30(4): 30–36.

Despite its popularity, definitions and concepts for PPPs are often contested, and no single definition of a PPP exists in the field of nutrition (Roehrich et al., 2014; Fanzo et al., 2021). In line with an Institute of Medicine (IOM) report, this review embraced a wide range of partnering arrangements (Institute of Medicine, 2012). The IOM emphasized the value of involving all sectors in working together to tackle complex public health problems, such as obesity, including government, academia, industry, and nongovernmental organizations (Institute of Medicine, 2012). Figure 1 shows the conceptual model used to define PPPs in this review. The public sector is comprised of national and subnational governments and governmental services. The private sector is divided into the business sector and the nongovernmental/academia sectors, broadly aligning with the “for-profit” and “nonprofit” delineation (Bajracharya and Hastings, 2015).

## Methods

### Study design

This review follows the methodological guidelines of a systematic literature review (Tufanaru et al., 2017). Systematic literature reviews seek to provide a comprehensive, unbiased synthesis of studies on a particular topic through a systematic and transparent approach (Tufanaru et al., 2017). Based on the study objective, the following Participants, Intervention, Comparison, and Outcome (PICO) framework (O’Connor et al., 2008) was developed to guide the review:

**Population**: Healthy school-aged children, defined as >5 and <18 years of age.

**Intervention/Exposure**: Contact with an intervention, program, or service that is part of a PPP.

**Comparator**: None.

**Outcome**: Changes in fruit and vegetable (F&V) consumption, changes in sugar-sweetened beverage (SSB) consumption, changes in energy-dense food consumption, or changes in body mass index (BMI).

### Inclusion and exclusion criteria

Articles were eligible for inclusion if they met the PICO criteria above. Studies were excluded if they were not available in English, were not published in peer-reviewed journals, and were not primary research studies.

Healthy school-aged children were defined as those with an absence of complex or chronic diseases based on clinical signs and symptoms. However, given the prevalence of overweight and obesity, studies inclusive of children with overweight or obesity in the absence of other complex chronic diseases were included. Studies focused exclusively on children aged less than 5 years were excluded, as the definition of obesity changes for this age group (World Health Organization [WHO]). PPPs were defined as any partnering agreement between a public and private sector entity as described in the study design section. Studies that did not include sufficient description to allow for partners’ identification were excluded. As studies evaluating PPPs were unlikely to have a comparator or control group, none were required for inclusion.

Primary outcomes were changes in F&V consumption, SSB consumption, or energy-dense food consumption, as these are known diet-related risk factors for childhood obesity (Poorolajal et al., 2020) and food consumption behaviors applicable to both high- and low-income countries. Studies measuring changes in access to or acquisition of these food groups were included as these are proxy measures of consumption. Studies examining only non-dietary obesity risk factors (e.g. physical activity, sedentary behavior, sleep duration, etc.) were beyond the scope of this review and therefore excluded. Studies reporting only changes in BMI were included if any of the previous diet-related risk factors were described as being targeted through the PPP intervention, but only BMI measures were reported as outcomes within the study.

### Search strategy and study selection

A search for articles between January 1990 and December 2021 was performed on 3 January 2022 using the electronic databases MEDLINE, PsycInfo, and Web of Science. These three databases encompass a wide range of research across natural sciences, health sciences, and social sciences, providing an extensive overview of published literature in the fields of public health, childhood obesity, and behavior change interventions. The start date for the search was based on previous reviews demonstrating PPPs rarely appeared in the literature before this time point (Barr, 2007; Roehrich et al., 2014). The search was undertaken with a uniform set of search terms, along with Boolean logic modified to the select database (Supplementary Table 1).

**Figure 2.**
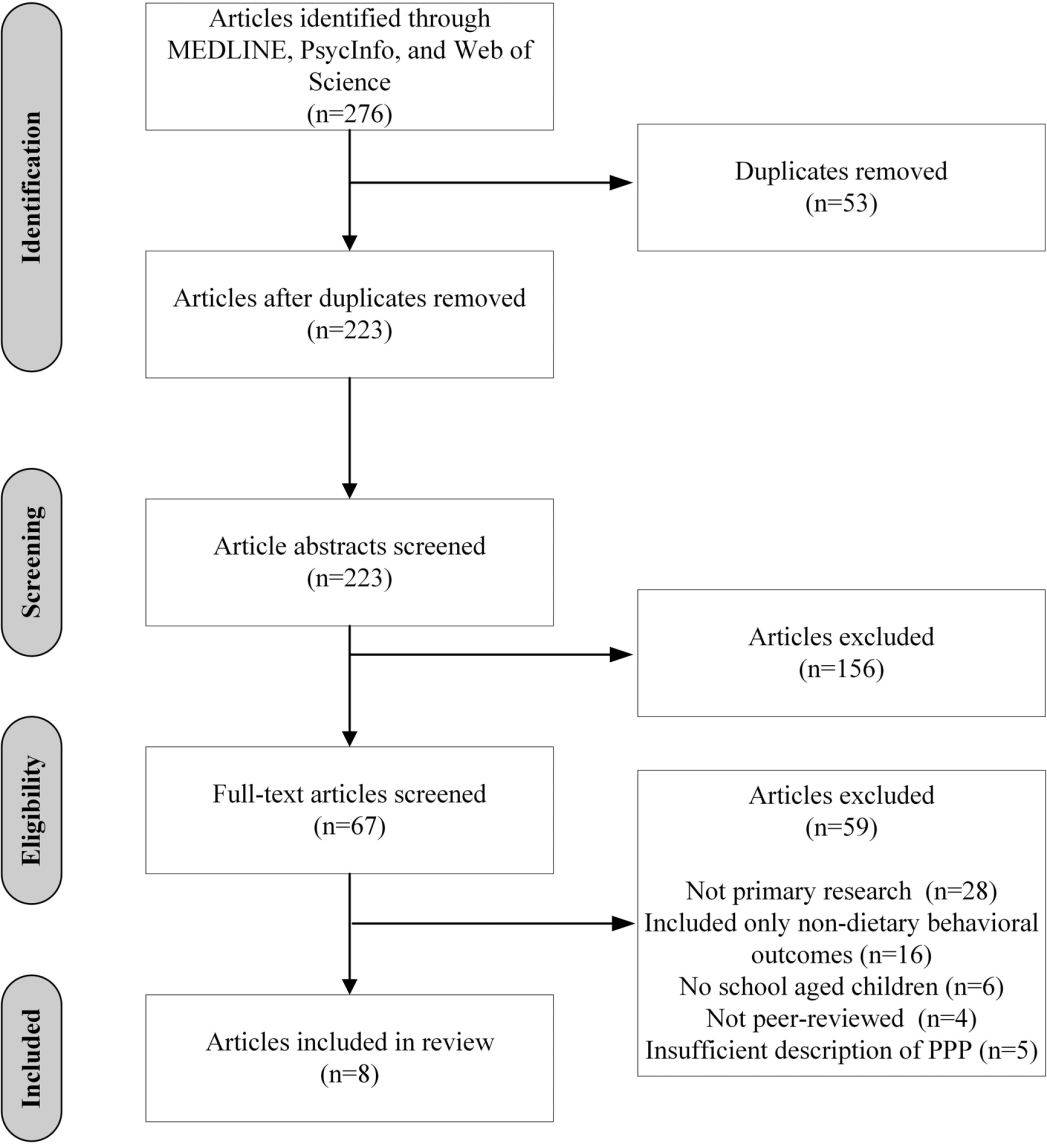
PRISMA diagram.

One reviewer undertook both the abstract and full-text screening. Ten percent of full-text articles were double-screened to ensure intra-rater reliability. The database search resulted in 276 articles. After removing duplicates, 223 studies remained. The initial round of title and abstract screening yielded 67 eligible articles. A further round of full-text screening resulted in eight articles for inclusion in this review (Figure 2). All screening was undertaken using the Covidence systematic review software (Veritas Health Innovation, 2021).

### Data extraction and analysis

Articles included in this review were analyzed and data were extracted for details on the following categories: study characteristics (publication year, country of study, study design, research aims, intervention, study participants, relevant risk factors targeted, and critical appraisal), partners involved (partner names and partner sectors), description of partnerships (partnership objectives and partnering arrangements), and partnership outcomes (effects on diet-related obesity risk factors). All data were collected, stored, and analyzed in Microsoft Excel.

A critical appraisal of study quality was undertaken for each of the included studies using the Quality Assessment Tool for Quantitative Studies from the Effective Public Health Practice Project (EPHPP) (Thomas et al., 2004). Overall study quality was assessed based on eight criteria: selection bias, study design, confounders, blinding, data collection methods, withdrawals and dropouts, intervention integrity, and analyses. Global ratings of strong, moderate, or weak were calculated following EPHPP scoring guidelines.

Given the heterogeneity of study designs related to PPPs and childhood obesity prevention and management, the effect of PPPs on diet-related obesity risk factors was evaluated on a descriptive basis, rather than quantitatively. Following data extraction and critical appraisal, data were organized according to the type of partnership and frequency counts were calculated for specific study attributes.

## Results

### Characteristics of studies included in the review

This review included 8 primary research studies. All studies took place in the United States and were published between 2010 and 2017. A summary of study characteristics is available in Table 1. Most studies were cohort studies (75%; n = 6), apart from one quasi-experimental study and one case study. Study participants ranged in age from approximately 5–17 years of age, with 50% of studies targeting low-income children. Three studies targeted only children with overweight or obesity.

**Table 1.** Description of studies included in the review.

Author	Country of study	Study design	Study aims	Intervention	Intervention setting	Target population	Risk factors targeted	Critical appraisal^a^
Cluss et al. (Cluss et al., 2010)	United States	Cohort study	To evaluate the feasibility of delivering a pediatric weight management intervention for low-income children	8-week structured weight management program delivered through weekly sessions of 30–45 min focused on nutrition education, physical activity, and behavior change.	Healthcare clinic and children's homes	Fifty-two children aged 4–12 years with overweight or obesity	F&V consumption; energy-dense food consumption; SSB consumption	Moderate
Fagan et al. (Fagan et al., 2010)	United States	Cohort study	To evaluate the implementation of a school-based obesity prevention program offered within an elementary school.	10-week structured healthy lifestyle program focused on nutrition education, physical activity, and life skills	Primary school	Eight-hundred seventy eight children in grades kindergarten through 6th grade with overweight or obesity	F&V consumption; SSB consumption	Weak
Harris et al. (Harris et al., 2012)	United States	Case study	To describe a public-private partnership to promote and sponsor school salad bars in low-income schools.	Provision of free or subsidized salad bar packages to schools	Primary and secondary schools	Children in school grades kindergarten through grade 12	F&V consumption	Weak
Springer et al. (Springer et al., 2012)	United States	Quasi- experimental study	To assess the impact of a healthy lifestyle program on physical activity, F&V consumption, and related psychosocial factors among low-income elementary school children.	6-month structured healthy lifestyle program focused on healthy eating and physical activity	Primary schools	Three-hundred eighty-three children in school grades 4 and 5	F&V consumption	Weak
Mitchell et al. (Mitchell et al., 2013)	United States	Cohort study	To evaluating weight changes in children referred to a weight management program	12-week structured weight management program delivered through weekly sessions of 30-min focused on healthy eating and physical activity	Healthcare clinic	Two-hundred eighty children aged 10–17 years with overweight or obesity	F&V consumption; energy-dense food consumption; SSB consumption	Weak
Mason et al. (Mason et al., 2014)	United States	Case study	To evaluate the acceptability, sales impact, and implementation barriers for a healthier snack vending initiative.	Placement of new vending machines meeting healthy nutrition standards throughout public park facilities	Parks and recreation facilities	Children and adults visiting the park system	Energy-dense food consumption	Moderate
Gorham et al. (Gorham et al., 2015)	United States	Cohort study	To assess the effect of a discount produce distribution program on F&V consumption among low-income children.	5-month market intervention with discounted produced delivered to six community sites	Community-based organizations (unspecified)	Four-hundred eighty children aged 3–13 years	F&V consumption	Moderate
Pierce et al. (Pierce et al., 2017)	United States	Cohort study	To describe and evaluate the implementation of the integrative health intervention through a public-private partnership.	6-week healthy life skills building program delivered daily for 6.5 h focused on nutrition education, physical activity, and leadership	Farm	Thirty-six children in school grades 9 and 10	F&V consumption; energy-dense food consumption	Moderate

^a^ Critical appraisal is based on the Effective Public Health Practice Project (EPHPP) (Thomas et al., 2004).

BMI: body mass index; F&V: fruit & vegetable; SSB: sugar-sweetened beverage.

The majority of studies (62.5%; n = 5) included structured, healthy lifestyle interventions, with varying degrees of intensity and duration. The remaining interventions focused on changes to children's food environment. Schools were the most frequently cited setting for delivering interventions (37.5%; n = 3). Nearly all studies included intervention components aimed at behavior change around F&V consumption (88%; n = 7), followed by energy-dense food consumption (50%, n = 4), and SSB consumption (38%; n = 3). A critical appraisal of study quality found half of the studies were of moderate quality and half were of weak quality based on the EPHPP tool (Thomas et al., 2004).

### Partnerships and partnering outcome of studies included in the review

A description of the partnerships undertaken to address diet-related obesity risk factors is listed in Table 2. All studies included both public sector and business sector partners. Most studies included partnerships across all three sectors: public sector, business sector, and nongovernmental/academic sectors (75%; n = 6). Public sector partners were most often state Medicaid programs (25%; n = 2), federal food assistance programs (25%; n = 2), and public schools (25%; n = 2). Other public sector partners included public parks and an elected government office. Business sector partners tended to be food producers (25%; n = 2), food retailers (25%; n = 2), and private healthcare providers (25%; n = 2). Other business sector partners included a private health insurance plan and a commercial weight loss program. Out of the six studies that included nongovernmental/academic sector partners, the most common partners were philanthropic organizations and academic research centers.

**Table 2.** Description of partnerships and partnering outcomes included in review.

Partnership type	Author	PPP initiative Name	PPP initiative purpose	Names of partners involved	Partnership arrangement	Key outcomes
Public sector with business sector	Mitchell et al. (Mitchell et al., 2013)	TennCare Weight Watchers Partnership Program	To help treat obesity in Tennessee by allowing pediatric Medicaid recipients to attend Weight Watchers with no out-of-pocket cost	**Business Partner(s):** Weight Watchers International **Public Partner(s):** TennCare **Nongovernmental/Academic Partner(s):** None	Contractual: Behavior change program delivery; Program delivery and participation costs	Participants who attended the program for ≥10 meetings had a 5% decrease in BMI z-scores
	Cluss et al. (Cluss et al., 2010)^a^	Healthy Eating and Activity for Life Time Habits (HEALTH)	To adapt and deliver an evidence-informed pediatric obesity intervention for Medicaid-insured families	**Business Partner(s):** Pediatric primary care practices **Public Partner(s):** Pennsylvania Medicaid **Nongovernmental/Academic Partner(s):** None	Contractual: Behavior change program delivery; Evaluation funding	81% of families reported eating fewer low nutrient, energy-dense foods ≥3 times per week and 88% reported eating high-nutrient, lower-energy foods. No changes in children's BMI z-scores.
Public sector with business sector and nongovernmental sector	Springer et al. (Springer et al., 2012)	Marathon Kids	To build partnerships with schools, community leaders and the private sector to promote physical activity and F&V consumption among children in grades kindergarten through grade 5 and their families	**Business Partner(s)**: Not specified **Public Partner(s)**: Public elementary schools in Texas **Nongovernmental/Academic Partner(s)**: Marathon Kids	Unspecified: Behavior change program delivery; Program delivery and participation costs	Students attending intervention school reported greater frequency of consuming fruit (3.25 vs. 2.96, *p* = 0.008) and vegetables (2.67 vs. 2.38, *p* = 0.008) per day compared to control school. No significant changes were found in weight.
	Harris et al. (Harris et al., 2012)	Lets Move Salad Bars to Schools (LMSB2S)	To raise awareness of the importance of school salad bars for improving child nutrition and to place 6000 salad bars in schools over 3 years	**Business Partner(s):** National Fruit & Vegetable Alliance; Whole Foods Market **Public Partner(s):** National School Lunch Program **Nongovernmental/Academic Partner(s):** United Fresh Produce Association Foundation; Food Family Farming Foundation	Unspecified: Donation pledges (financing and infrastructure); Discounted procurement arrangements	Two years after project launch, the initiative had delivered >1400 salad bars to schools and given >700,000 school children increased access to F&V in school cafeterias.
	Pierce et al. (Pierce et al., 2017)^a^	Mission Thrive Summer (MTS)	To provide youth with integrative health education by engaging them in directly in healthy self-care practices and life skills under the guidance of positive role models, with the goal of motivating long-term healthy behaviors	**Business Partner(s):** Real Food Farms; Maryland Institute for Integrative Health **Public Partner(s):** Baltimore City Mayor's Office **Nongovernmental/Academic Partner(s):** University of Maryland School of Medicine	Unspecified: Preferential employment opportunities to program participants; Minimum wage agreement for program participants; Shared-space infrastructure.	No changes were found in F&V intake during Year 1. New measurement tool in Year 2 found an increase in participants frequency of consumption of tomatoes (*p* = 0.01), other vegetables (*p* = 0.02), and a decrease in frequency of consumption of hot dogs, corn dogs or sausage (*p* = 0.0003), hamburgers or cheese burgers (*p* = 0.0006), and ice cream (*p* = 0.01)
	Mason et al. (Mason et al., 2014)^a^	Chicago Park District's 100% Healthy Vending Initiative	An initiative to strengthen and support healthful vending efforts throughout the Chicago parks system	**Business Partner(s):** Compass Group (vending) **Public Partner(s):** Chicago Park District **Nongovernmental/Academic Partner(s):** Robert Wood Johnson Foundation	Contractual: Vending contract	The initiative placed 98 snack vending machines throughout the park system. 100% of items sold met nutrition standards and were uniformly priced. 77% of observed purchases in the machines were made by children and adolescents.
	Fagan et al. (Fagan et al., 2010)	Dare to Live Healthy the Red Clay Way	A partnership to implement an evidence-based, multicomponent obesity prevention program	**Business Partner(s):** Blue Cross Blue Shield of Delaware **Public Partner(s):** Brandywine Springs Elementary School **Nongovernmental/Academic Partner(s):** Cristiana Care Health System	Unspecified	Participants reported improvements in F&V consumption (*p* < 0.01) and drinking high sugar beverages (*p* < 0.01) compared to baseline. At follow-up 30 children in the overweight category had moved into normal weight and 21 children in the obese category had moved to overweight.
	Gorham et al. (Gorham et al., 2015)^a^	Fresh to You	To bring discount fresh produce markets to low-income neighborhoods	**Business Partner(s):** Local distributor of F&Vs (unspecified) **Public Partner(s):** Supplemental Nutrition Assistance Program **Nongovernmental/Academic Partner(s):** Brown University	Unspecified: Discounted procurement arrangement; Market access agreement	Children's consumption increased for fruit without juice (0.20 cups, *p* = 0.003), vegetables without potatoes (0.28 cups, *p* = 0.03), and F&Vs combined (0.48 cups, *p* < 0.01).

^a^ Studies determined to be of Moderate quality based on the Effective Public Health Practice Project (EPHPP) (Thomas et al., 2004).

BMI: body mass index; F&V: fruit & vegetable; PPI, public–private partnership; SSB: sugar-sweetened beverage.

Most partnering arrangements described in the studies were contractual arrangements (38%; n = 3), with two focusing on the delivery of a structured healthy lifestyle intervention and one on a vending contract. However, the majority of studies (62%; n = 5) did not specify the legal mechanism underpinning their partnership and provided limited descriptions of the products or services provided by different partners. Some of these partnering arrangements included discounted procurement arrangements, shared-space infrastructure, preferential employment opportunities, donation pledges, and market access agreements.

The small sample size and study heterogeneity limits comparisons across studies. Interventions varied by intervention components, duration, delivery settings, and populations targeted. Of the four studies with moderate quality ratings, three found decreases in energy-dense food consumption or the availability of energy-dense foods. One study showed significant increases in F&V consumption between baseline and follow-up of a 5-month healthy lifestyle intervention. Only one moderate quality study measured changes in BMI, and no difference was found in BMI z-score between baseline and follow-up. Of the four studies with weak quality ratings, three found increases in F&V consumption or availability of F&Vs. Three studies included outcome BMI measures, of which two found decreases in BMI at follow-up. The one study that reported on changes in SSB consumption found significant decreases in SSB consumption following a 10-week healthy lifestyle intervention.

## Discussion

Childhood obesity is a serious and costly public health problem, driven by multiple and interacting genetic, behavioral, social, and environmental factors. Novel approaches and new structures, including PPPs, are currently promoted as mechanisms to address the complex nature of the challenge (Institute of Medicine, 2015; Institute of Medicine, 2012; Hendriks et al., 2013).

To the author's knowledge, this is the first systematic literature review to describe the effects of interventions carried out through PPPs on diet-related obesity risk factors among school-aged children. The results suggest that certain PPPs may be beneficial mechanisms for addressing diet-related obesity risk factors among school-aged children. However, the small sample size and heterogeneity in study design, target populations, intervention components, settings, and risk factors targeted all limit comparisons. Moreover, few studies described the financial or legal arrangements that governed their partnerships, much less the challenges to implementing interventions within these legal or financial frameworks. While all studies were of moderate to weak quality, those which reported on outcomes relevant to this review found either no changes or beneficial changes in diet-related obesity risk factors.

When it comes to issues of public health, engagement between the public and private sectors, particularly the business sector, is often complex and contentious. Valid concerns exist as to whether commercial interest can be balanced with public health goals or whether the risk to public institutions’ credibility outweighs any potential benefit of partnering (De Pinho Campos et al., 2019). This review found business sector partners participating in PPPs were primarily food producers, food retailers, and private health care providers. No PPPs with food manufacturers (i.e. those responsible for processing raw ingredients into packaged, edible food products) were identified. This may reflect a risk reduction strategy on the part of public sector actors in opting to partner with only certain actors within the food industry. The food industry is not one monolithic group, but encompasses all the actors involved in food production, processing, distribution, consumption, and ultimately disposal. Partnerships with those who produce food (e.g., Fruit and Vegetable Growers’ Association) and those who retail a wide range of food products (e.g., grocery store chains) may carry inherently less risk to public partnerships than those who manufacture highly processed foods and beverages. Policy makers interested in partnering with actors in the food industry may benefit from developing a conflicts of interest policy, using risk-based and due diligence approaches to identify conflicts of interests, and establishing a framework for managing these conflicts. Some guidance tools are readily available through the Scaling Up Nutrition Movement (Scaling Up Nutrition Movement, 2014), the WHO (Ralston et al., 2020), and the Ontario Ministry of Health (Kraak, 2014).

While this review sought to describe and characterize the different partners involved in PPPs and their partnering arrangements, this was challenging on several fronts. First, a high level of ambiguity exists in classifying partners as either “public” or “private”. For organizations like healthcare systems and schools, country-specific context often determines whether an entity is “public” or “private”, making classification and comparison across contexts challenging. Philanthropic organizations, largely classified as “nonprofits” and excluded from research on PPPs, can still have questionable ties to commercial interests. For example, the CDC Foundation, and subsequently the WHO Foundation, have both received criticism for being pass-through nonprofits, allowing their namesake organizations to receive industry funding that would otherwise have been prohibited due to conflicts of interest (Lenzer, 2015; Maani et al., 2021). The current review embraced a broad definition of “private” in an attempt to address these nuances by including both for-profit and nonprofit private organizations. Even with a broader definition, a strictly binary classification of “public” versus “private” is likely limiting. Future efforts aimed at developing a comprehensive taxonomy to classify the different partnering mechanisms across sectors would greatly benefit the field of PPP research. Finally, very few studies included in this review provided details on the governance structure, legal, or financial mechanisms underpinning the PPPs. Partnership arrangements span a continuum ranging from highly formalized agreements (e.g., legislated or formal contracts) to informal agreements (Bajracharya and Hastings, 2015). Understanding these arrangements can shed light on power dynamics, resource contributions, accountability mechanisms, etc. Public sector funders can improve the transparency of partnering arrangements by requiring these to be disclosed for all publicly funded grant recipients.

## Limitations

This systematic literature review had several limitations. First, the outcomes of interest were narrow and excluded other known diet-related obesity risk factors, such as eating breakfast, family mealtimes, and alcohol consumption (Poorolajal et al., 2020). These risk factors were excluded because they are often culturally specific, while F&V consumption, SSB consumption, and energy-dense food consumption were likely to be applicable across contexts. Second, the literature databases and keyword search string likely limited the search results. Given the complex nature of PPPs, terminology is often inconsistent when describing these relationships. The keywords used may not have been sufficiently broad enough to capture many studies involving both public and private partners. Finally, the studies ultimately included in this review all originated from the United States. This finding may reflect unconscious selection bias on the part of the reviewer or context-specific terminology used in the search string.

## Conclusion

Childhood obesity is a serious and complex public health problem, driven by many compounding risk factors, including unhealthy diets. It has been argued that collaboration across all sectors, including the private sector, is needed to address the drivers of obesity in places children live, learn, and play. Nevertheless, little is known about the different types of PPPs undertaken to address diet-related obesity risk factors nor what effect interventions carried out through these partnerships have on school-aged children. This review synthesizes the evidence on PPPs pertaining to diet-related risk factors for childhood obesity. While all studies were of moderate to weak quality, the results reveal either no changes or beneficial changes in children's F&V consumption, SSB consumption, energy-dense food consumption, or BMI. Business sector partners largely represented food producers, food retailers, and private healthcare providers. Few studies described the financial or legal arrangements that governed their partnerships. Given the ambiguity around defining PPPs, future research efforts are needed to develop a taxonomy for better identifying and classifying the different partnering arrangements that can exist between public and private sector partners to improve public health.

## Supplemental Material

sj-docx-1-nah-10.1177_02601060221136184 - Supplemental material for Effects of public–private partnership on diet-related obesity risk factors among school-aged children: A systematic literature review Click here for additional data file. Supplemental material, sj-docx-1-nah-10.1177_02601060221136184 for Effects of public–private partnership on diet-related obesity risk factors among school-aged children: A systematic literature review by Megan R Harrison in Nutrition and Health
