# Supplementary material for: Effects of public–private partnership on diet-related obesity risk factors among school-aged children: A systematic literature review
Source: Nutr Health. 2022 Nov 4;29(3):453–63. doi: 10.1177/02601060221136184 (PMC10503256; doi:10.1177/02601060221136184)
Supplement: sj-docx-1-nah-10.1177_02601060221136184 - Supplemental material for Effects of public–private partnership on diet-related obesity risk factors among school-aged children: A systematic literature review [file sj-docx-1-nah-10.1177_02601060221136184.docx]

## Supplementary Table 1. Search Strategies

| **MEDLINE** | | |
| --- | --- | --- |
| 1 | exp Public-Private Sector Partnerships/ | 2383 |
| 2 | (Commercial partnership* or collaborative governance or business partnership* or PPP* or market based approach* or industry partnership* or private public partnership* or public private partnership* or private public participation or public private engagement or public private mix or public private relationship* or inter organization* or public non-profit private enterprise* or public alliance* or non-profit partnership* or nonprofit partnership* or private partnership*).mp. | 15053 |
| 3 | 1 or 2 | 16713 |
| 4 | exp Obesity/ | 237364 |
| 5 | Weight Gain/ | 33450 |
| 6 | (obes* or overweight or over weight).mp. | 414962 |
| 7 | ((bmi or body mass index) adj2 (gain or change or increase)).mp. | 7400 |
| 8 | 4 or 5 or 6 or 7 | 442498 |
| 9 | exp Sugar-Sweetened Beverages/ | 597 |
| 10 | ((sugar* or carbonated or fizzy or energy or soft or sport* or sweet* or pop) adj2 (drink* or beverage* or soda* or milk)).mp. | 16582 |
| 11 | (SSB* or cola).mp. | 9298 |
| 12 | ((tea or coffee or milk or dairy or water or soda) adj2 (flavo?r* or sugar* or sweet*)).mp. | 3917 |
| 13 | 9 or 10 or 11 or 12 | 26076 |
| 14 | exp Fruit/ | 115528 |
| 15 | exp Vegetables/ | 33860 |
| 16 | (fruit or vegetable*).af. | 164331 |
| 17 | (Apple* or pear* or grape* or banana* or berr* or citrus or blackberr* or blueberry* or cranberry* or guava* or kiwi* or lingonberr* or mango* or melon* or papaya* or raspberr* or tangerine* or plum or grapefruit* or strawberr*).mp. | 168592 |
| 18 | (Carrot* or greens or cabbage* or brassica* or celery or spinach* or salad* or pea or peas or bean or beans or onion* or broccoli or cauliflower or beetroot* or turnip* or rhubarb or potato* or tomato*).mp. | 153256 |
| 19 | 14 or 15 or 16 or 17 or 18 | 488761 |
| 20 | exp Fast Foods/ | 2665 |
| 21 | (Ultra processed or ultraprocessed or ready to eat or ready to consume or fast food* or junk food* or snack* or unhealthy diet* or unhealthy food*).mp. | 21091 |
| 22 | ((calorie* or energy or kilocalorie*) adj2 (high* or dense)).mp. | 72243 |
| 23 | 20 or 21 or 22 | 92096 |
| 24 | exp adolescent/ or exp child/ | 3216904 |
| 25 | (child* or schoolchild* or preschool* or pre school* or kindergarten* or schoolage* or school age* or schoolboy* or schoolgirl* or boy* or girl* or preteen* or teen* or adolescen* or youth* or young people or young person* or p?ediatr*).mp. | 3905638 |
| 26 | 24 or 25 | 3905638 |
| 27 | 8 or 13 or 19 or 23 | 1009635 |
| 28 | 3 and 26 and 27 | 96 |
| 29 | limit 28 to yr="1990 - 2021" | 96 |
| **PsycInfo** | | |
| 1 | exp Public Sector/ and exp Private Sector/ | 1596 |
| 2 | (Commercial partnership* or collaborative governance or business partnership* or PPP* or market based approach* or industry partnership* or private public partnership* or public private partnership* or private public participation or public private engagement or public private mix or public private relationship* or inter organization* or public non-profit private enterprise* or public alliance* or non-profit partnership* or nonprofit partnership* or private partnership*).mp. | 2576 |
| 3 | exp Obesity/ | 26572 |
| 4 | Weight Gain/ | 3349 |
| 5 | (obes* or overweight or over weight).mp. | 51738 |
| 6 | ((bmi or body mass index) adj2 (gain or change or increase)).mp. | 1260 |
| 7 | 3 or 4 or 5 or 6 | 53793 |
| 8 | exp "beverages (nonalcoholic)"/ | 2078 |
| 9 | ((sugar* or carbonated or fizzy or energy or soft or sport* or sweet* or pop) adj2 (drink* or beverage* or soda* or milk)).mp. | 3095 |
| 10 | (SSB* or cola).mp. | 930 |
| 11 | ((tea or coffee or milk or dairy or water or soda) adj2 (flavo?r* or sugar* or sweet*)).mp. | 815 |
| 12 | 9 or 10 or 11 or "15".mp. [mp=title, abstract, heading word, table of contents, key concepts, original title, tests & measures, mesh word] | 200205 |
| 13 | exp Fruit/ | 4375 |
| 14 | (fruit or vegetable*).af. | 31900 |
| 15 | (Apple* or pear* or grape* or banana* or berr* or citrus or blackberr* or blueberry* or cranberry* or guava* or kiwi* or lingonberr* or mango* or melon* or papaya* or raspberr* or tangerine* or plum or grapefruit* or strawberr*).mp. | 25783 |
| 16 | (Carrot* or greens or cabbage* or brassica* or celery or spinach* or salad* or pea or peas or bean or beans or onion* or broccoli or cauliflower or beetroot* or turnip* or rhubarb or potato* or tomato*).mp. | 4297 |
| 17 | 14 or 15 or 16 or "20".mp. or "21".mp. [mp=title, abstract, heading word, table of contents, key concepts, original title, tests & measures, mesh word] | 376941 |
| 18 | exp Fast Food/ | 308 |
| 19 | (Ultra processed or ultraprocessed or ready to eat or ready to consume or fast food* or junk food* or snack* or unhealthy diet* or unhealthy food*).mp. | 6229 |
| 20 | ((calorie* or energy or kilocalorie*) adj2 (high* or dense)).mp. | 2991 |
| 21 | ("23" or "24" or "25").mp. | 330963 |
| 22 | exp Pediatrics/ | 32577 |
| 23 | (child* or schoolchild* or preschool* or pre school* or kindergarten* or schoolage* or school age* or schoolboy* or schoolgirl* or boy* or girl* or preteen* or teen* or adolescen* or youth* or young people or young person* or p?ediatr*).mp. | 1210509 |
| 24 | 7 or 12 or 17 or 21 | 821746 |
| 25 | 1 or 2 | 4059 |
| 26 | 22 or 23 | 1213693 |
| 27 | 24 and 25 and 26 | 88 |
| 28 | limit 27 to yr="1990 - 2021" | 84 |
| **Web of Science** | | |
| 1 | TS=("Commercial partnership*" or "collaborative governance" or "business partnership*" or "PPP*" or "market based approach*" or "industry partnership*" or "private public partnership*" or "public private partnership*" or "private public participation" or "public private engagement" or "public private mix" or "public private relationship*" or "inter organization*" or "public non-profit public enterprise*" or "public nonprofit public enterprise*" or "public alliance*" or "non-profit partnership*" or "nonprofit partnership*" or "private partnership*") | 34336 |
| 2 | TS=("obes*" or "overweight" or "over weight") | 489236 |
| 3 | TS=(("bmi" or "body mass index") NEAR/2 ("gain" or "change" or "increase")) | 9181 |
| 4 | #2 OR #3 | 492284 |
| 5 | TS=(("sugar*" or "carbonated" or "fizzy" or "energy" or "soft" or "sport*" or "sweet*" or "pop") NEAR/2 (drink* or beverage* or soda* or milk)) | 23442 |
| 6 | TS=(SSB* or cola) | 238515 |
| 7 | TS=((tea or coffee or milk or dairy or water or soda) NEAR/2 (flavo$r* or sugar* or sweet*)) | 9966 |
| 8 | #5 OR #6 OR #7 | 267160 |
| 9 | TS=(fruit or vegetable*) | 393153 |
| 10 | TS=(Apple* or pear* or grape* or banana* or berr* or citrus or blackberr* or blueberry* or cranberry* or guava* or kiwi* or lingonberr* or mango* or melon* or papaya* or raspberr* or tangerine* or plum or grapefruit* or strawberr*) | 437074 |
| 11 | TS=(Carrot* or greens or cabbage* or brassica* or celery or spinach* or salad* or pea or peas or bean or beans or onion* or broccoli or cauliflower or beetroot* or turnip* or rhubarb or potato* or tomato*) | 976979 |
| 12 | #9 OR #10 OR #11 | 1620199 |
| 13 | TS=("Ultra processed" or ultraprocessed or "ready to eat" or "ready to consume" or "fast food*" or "junk food*" or "snack*" or "unhealthy diet*" or "unhealthy food*") | 29000 |
| 14 | TS=((calorie* or energy or kilocalorie*) NEAR/2 (high* or dense)) | 335894 |
| 15 | #13 OR #14 | 363392 |
| 16 | TS=(child* or schoolchild* or preschool* or "pre school*" or kindergarten* or schoolage* or "school age*" or schoolboy* or schoolgirl* or boy* or girl* or preteen* or teen* or adolescen* or youth* or "young people" or "young person*" or "p$ediatr*") | 2687010 |
| 17 | #4 OR #8 OR #12 OR #15 | 2668130 |
| 18 | #1 AND #16 AND #17 | 96 |
